# Do healthier lifestyles lead to less utilization of healthcare resources?

**DOI:** 10.1186/s12913-017-2185-4

**Published:** 2017-03-31

**Authors:** I-Chen Lee, Chao-Sung Chang, Pey-Lan Du

**Affiliations:** 1 Department of Healthcare Administration and Medical Informatics, Kaohsiung Medical University, 100, Shih-Chuan 1st Road, Kaohsiung, 807 Taiwan; 2 Department of Hematology-Oncology, E-Da Cancer Hospital; School of Medicine for International Students, I-Shou University, No.21, Yi-Da Road, Jiao-Su Village, Yan-Chao District, Kaohsiung City, 824 Taiwan; 3 Department of Sport and Leisure, National Quemoy University, No.1, Daxue Rd., Jinning Township, Kinmen County, Kinmen, 892 Taiwan

**Keywords:** Utilization of healthcare resources, Healthy lifestyle, Preventive health services

## Abstract

**Background:**

Governments are urged to determine methods to control the use of medical resources and curb the rise of healthcare costs. The question is, do health behaviors have an impact on the use of medical resources? This study aims to identify and understand the difference in the number of outpatient visits and health examinations based on various health behaviors and to determine whether patients seek medical care for illness from the same physicians.

**Methods:**

This study used the dataset derived from the Department of Budget, Accounting and Statistics of Kaohsiung, Taiwan in 2005. Persons older than 15 years were surveyed using an on-site questionnaire. A total of 2911 persons were enrolled in this study. Independent *t*-tests, chi-square tests, one-way ANOVA, multiple linear regression and binominal logistic regression were used in the data analysis.

**Results:**

The regression model for the frequency of doctor visits, health examinations, and whether the same physician is sought for medical care has demonstrated significant correlations with gender, age and education-level variables. Four health behaviors (i.e., exercise habits, dietary habits, regular blood pressure measurement, drinking habits) exhibited a significant correlation with healthcare utilization (*P* <0.05).

**Conclusions:**

Healthy lifestyles lead to an increase in the utilization of preventive health services. However, there is not much significantly reducing the number of outpatient visits in people with health behaviors. Specifically, people with regular exercise habits and who take their blood pressure measurement regularly have an increased number of outpatient visits. It is suggested that more available and accessible health consultation services be provided to inculcate in the general public the importance of maintaining a healthy lifestyle.

## Background

There is a steady increase in the utilization of national healthcare resources because of the aging population in recent years, along with the rapid increase in healthcare costs. Governments are urged to determine methods to control the use of healthcare resources and curb the rise of healthcare costs [1–3]. Healthcare resources include medical-related services (i.e., outpatient visits, emergency care, and hospitalization) and preventive services (i.e., health examinations). Usually, medical-related and preventive services are both provided by professionals from general hospitals or clinics, however, the goals of thoses two services are different. People receive medical-related services to relieve the discomfort or to slow down the progress of the disease when they have obvious physical symptoms. In contrast, preventive services are provided to ensure that healthy people who do not suffer from any physical discomfort and do not need any specific treatments, or people who are at high-risk of having certain diseases don’t get sick. It is also done to detect early any symptoms of diseases. The research indicates that the probability of being hospitalized and the number of outpatient or emergency visits decreases as more preventive healthcare services are provided, and this may reduce healthcare costs [4–6].

Taiwan established the National Health Insurance in 1995; the policy eliminates the financial barriers encountered by most people during medical treatment and provides many preventive health-related services free of charge such as physical examinations for those over the age of 40. Meanwhile, the National Health Insurance Administration (NHIA) aggressively promotes the system of having a primary physician for family care since 2003 [7]. People are strongly suggested to seek medical care from the same physician when they are sick, so that the whole family history of health and diseases can be traced and monitored. In reference to the Western system of Family Medicine, people are encouraged to look for a reliable physician in the nearby community when they need primary medical care. The family physician may refer their clients to specific medical care specialists when advanced care is needed, and this physician will also obtain the necessary information or feedback through this referral system, which may enhance the preventive health care services and strengthen the network of primary medical care for the patients. As previously metioned, in order to slow down the increase in healthcare costs, it is necessary to provide adequate preventive healthcare services and to improve the utilization of these services. Most studies [8–11] have explored the utilization of healthcare resources, which focused on issues such as the frequency of visits to outpatient and emergency clinics, the number of patients admitted, and the length of stay for each hospitalization as well. However, there are few studies reviewing the utilization of preventive health care services and the related issues.

Andersen claimed that people’s health status is the most important determining factor in the utilization of health resources [12]. Some researchers [13–16] also reported that health conditions improve as people become more health conscious and maintain a healthy lifestyle, consequently, the use of medical resources decreases. Purvis (2013) [17] investigated the factors that influence health conditions, and the results showed that health behaviors have a significant effect on health conditions. Also, health behaviors were a major determinant of morbidity and mortality in the US [18]. Thus, the health behaviors, the health status, the utilization of medical resources, and the mortality rate for certain diseases are correlated significantly. Few individual studies provide comprehensive compilations of numerous health behaviors and consider them to be the main research topic. Most studies [19–21] have only addressed and explored on the risks of one or two health behaviors in relation to sickness, whereas the health behaviors have not been the main topic for the said studies.

In order to curb the rise of medical costs, the issues regarding the utilization of medical resources and preventive health care services shall be crucially addressed. In advance, this study aims to clarify what kinds of health behaviors might reduce the utilization of medical resources, and whether the health behaviors affect the utilization of preventive health care services as well. Thus, a survey on people’s health status in a specific region were conducted and analyzed in this study for two purposes: (1) to understand the relationship between health behaviors and the utilization of health care resources (i.e., the frequency of outpatient visits and health examinations), and (2) to explore how the specific type of health behaviors affect the utilization of health care resources.

## Methods

In 2005, an annual survey was conducted by the Department of Budget, Accounting and Statistics of Kaohsiung, using a well-designed and structured questionnaire, to study the correlation among lifestyles, utilization of health care resources, and the health status of the citizens in Kaohsiung. All questions derived from the literature were developed by the research committee and verified by a panel of professional experts. The panel of professional experts reached a 94% item-by-item agreement on content validity and appropriateness of wording.

Kaohsiung is the second biggest city in Taiwan. A two-stage stratified random sampling was used in this study. In the first stage, the townships were randomly selected from all the eleven administration districts in the city of Kaohsiung. In the second stage, the households were then randomly selected from the sampled townships. There were a total of 2911 people aged over 15 years in these sampled households who have been recruited as the subjects in this study. A trained interviewer was sent to assist the selected citizens to complete the questionnaire during face-to-face interviews. All the interviewers and researchers were fully oriented and highly trained in advance of the study for increased objectivity.

The secondary data analysis was conducted in the course of this study. The primary data were derived from the data base of the related government department. These use of data has been granted an exemption from requiring ethics approval according to the regulations set by the Ministry of Justice in Taiwan (http://mohwlaw.mohw.gov.tw/FLAW/FLAWDAT0202.aspx?lsid=FL066232). There are four types of variables in this study: demographic information, health behaviors, current health status, and utilization of health care resouces. Detailed descriptions of each type were provided as follows:

- Demographic information
  Demographic factors including sex, age, education level,and marital status were recorded.
- Health behaviors
  The respondents were provided with a list of dietary habits, exercise habits, behavioral habits such as smoking, drinking and betel nut chewing, and blood pressure measuring. The respondents then completed this questionnaire by truly reflecting their daily living habits and lifestyle. The dietary habits were recorded using 5 questions as to whether the respondents had the following habits: “eating breakfast”, “fixed number of meals and regular mealtime”, “5 varieties of vegetables or fruits a day”, “maintaining a low salt, low sugar and low fat diet”, and “being aware of the description and branding of food products”. The respondents were asked the following questions regarding health related behaviors: “Do you care about your own health condition?”, “Do you measure your blood pressure regularly?”, and “Have you exercised regularly in the past 3 months?” The habits of smoking, drinking and betel nut chewing were assessed by the questions, “Do you have a habit of smoking, drinking and betel-nut chewing?”
- Current health status
  The presence of any physician-diagnosed chronic illness was ascertained by the following question about each individual’s current health status: “Are you suffering from any chronic diseases including hypertension, diabetes, cardiovascular disease, musculoskeletal disease, respiratory disease, gout, and cancer?”
- Utilization of health care resources
  Information on the individual’s utilization of health care resources were obtained from the questions about the frequency of outpatient visits in the past 3 months and the utilization of preventive healthcare services, as well as the number of health exams taken in the past 3 years and whether individuals seek medical care for illness from the same physician. The footnote “disease-related outpatient visits” pertains to the question on the number of hospitals or clinics visited. The footnote, “disease-related examinations which were done in hospitals or clinics were excluded” pertains to the question on the number of health examinations taken. In the meanwhile, a list of all kinds of health examinations was provided as reference for clarification and to discern the utilization of these two types of health care resources. The number of health examinations has not been counted into the number of outpatient visits.
  The statistical analyses were performed using SPSS (Statistical Product and Service Solutions) software, version 19.0. Independent *t*-tests, chi-square tests and one-wayANOVA (analysis of variance) were used to examine the utilization of health care resources in terms of specific demographic characteristics, health status, and health behaviors. The demographic variables and health status were adjusted in multivariate analysis of regression to further clarify the association between health behaviors and the utilization of health care resources. A multiple linear regression model was performed to examine the association of health behaviors (independent variable), the frequency of outpatient visits (dependent variable), and the number of health exams taken (dependent variable). A binominal logistic regression model was used to determine the odds ratio among the groups of individuals seeking medical care for illness from the same physician (dependent variable). Additionally, the demographics and health status were considered as independent variables in the above regression model. In order to investigate the association between health behaviors and the utilization of health care resources more globally and comprehensively, all health behaviors were computed in the regression model. Simultaneously, the collinearity diagnosis was performed to assess whether the severity of association among health behaviors exist.

## Results

The data regarding the difference among the various demographic characteristics in the number of outpatient visits and health examinations and whether patients seek medical care for illness from the same physician is shown in Table 1. The results show that women seek medical treatment more frequently on a yearly basis and are more likely to seek medical care from the same physician. However, they undergo health examinations less frequently. Married individuals tend to go to outpatient visits and health examinations more frequently, and they prefer to be seen by the same physician for an illness. Scheffe's post hoc comparison indicates that people 45 years and older show a significant increase in the frequency of outpatient visits and health examinations; these patients have a greater chance of seeking medical care from the same doctor. Scheffe's post hoc comparison identifies that those with an elementary or lower education level tend to visit doctors more often, undergo health exams more frequently, and are more likely to visit the same doctor for illness. Those currently suffering from diseases frequently visit outpatient clinics, perform health examinations more often and receive medical care for illness from the same physician. These findings are statistically significant (*P* <0.05).

**Table 1:** The Association Between Demographic Characteristics And Healthcare Utilization

Variable	Number of outpatient visits		Number of health exams		Seen by the same physicians		
					Yes	No	
	Mean (SD)	*p*	Mean (SD)	*p*	*N* (%)	*N* (%)	*p*
Gender							
Male	0.92 (0.91)	<0.001	0.92 (1.33)	0.035	937 (46.3)	452 (50.8)	0.028
Female	1.05 (1.00)		0.82 (1.18)		1085 (53.7)	437 (49.2)	
Marital Status							
With Spouse	1.07 (0.99)	<0.001	1.00 (1.33)	<0.001	1251 (61.9)	484 (54.4)	<0.001
Without Spouse	0.88 (0.91)		0.67 (1.10)		771 (38.1)	405 (45.6)	
Age							
15–18years	1.50 (1.93)	<0.001	0.36 (0.78)	<0.001	138 (6.8)	94 (10.6)	<0.001
19–24years	1.90 (3.53)		0.47 (0.80)		162 (8.0)	90 (10.1)	
25–34years	1.75 (2.72)		0.62 (1.09)		283 (14.0)	146 (16.4)	
35–44years	2.26 (3.38)		0.72 (1.22)		347 (17.2)	187 (21.0)	
45–54years	2.62 (3.41)		0.98 (1.27)		476 (23.5)	174 (19.6)	
55–64years	3.93 (5.09)		0.92 (1.17)		301 (14.9)	112 (12.6)	
≧65 years	4.78 (4.98)		1.65 (1.61)		315 (15.6)	86 (9.7)	
Education Level							
Illiterate / Self Study	5.63 (6.31)	<0.001	1.29 (1.49)	<0.001	145 (7.2)	49 (5.5)	0.015
Elementary	4.00 (5.04)		1.01 (1.35)		317 (15.7)	111 (12.5)	
Junior High School	2.60 (3.58)		0.71 (1.20)		287 (14.2)	134 (15.1)	
Senior High School	2.23 (3.20)		0.73 (1.17)		656 (32.4)	339 (38.1)	
College/University	2.21 (3.05)		0.92 (1.26)		563 (27.8)	237 (26.7)	
Graduate& above	1.93 (2.10)		1.16 (1.35)		54 (2.7)	19 (2.1)	
Disease							
No	0.66 (0.74)	<0.001	0.67 (1.13)	<0.001	1146 (56.7)	627 (70.5)	<0.001
Yes	1.51 (1.04)		1.17 (1.37)		876 (43.3)	262 (29.5)	

Data from the structured questionnaire of the Department of Budget, Accounting and Statistics of Kaohsiung, Taiwan in 2005 weighted to be national representative

Further analyses indicate that health behaviors differ between the number of outpatient visits, the frequency of obtaining health exams, and seeking medical attention for illness from the same physician. Six health behaviors are related to health exams, whereas four are related to the frequency of outpatient visits and seeking medical care from the same physician (*P* < 0.05). For example, compared to those without regular exercise habits, people who exercise regularly visit a physician more frequently (mean = 1.12 v.s 0.91), they are more likely to take health exams (mean = 1.14 v.s 0.70), and have a higher probability of seeking the same physician for medical care (41.7%v.s31.6%). The remaining health behaviors, in relationship to the frequency of outpatient visits, health exams and seeking medical care from the same physicians, are noted in Table 2.

**Table 2:** The Association Between Individual Lifestyle and Healthcare Utilization

Variable	Number of outpatient visits		Number of health exams		Seen by the same physicians		
					Yes	No	
	Mean (SD)	*p*	Mean (SD)	*p*	*N* (%)	*N* (%)	*p*
Exercise Habits							
Yes	1.12 (1.01)	<0.001	1.14 (1.36)	<0.001	843 (41.7)	281 (31.6)	<0.001
No	0.91 (0.92)		0.70 (1.15)		1179 (58.3)	608 (68.4)	
Breakfast							
Yes	0.99 (0.97)	0.984	0.87 (1.25)	0.185	1966 (97.2)	862 (97.0)	0.781
No	0.99 (0.85)		0.69 (1.21)		56 (2.8)	27 (3.0)	
Regular meals							
Yes	0.99 (0.96)	0.905	0.88 (1.26)	0.001	1944 (96.1)	839 (94.4)	0.041
No	1.00 (1.04)		0.57 (1.03)		78 (3.9)	50 (5.6)	
5 servings of fruits& vegetables							
Yes	0.98 (0.95)	0.036	0.66 (1.13)	0.001	1807 (89.4)	782 (88.0)	0.295
No	1.10 (1.04)		0.89 (1.26)		215 (10.6)	107 (12.0)	
Dieting Habits							
Yes	0.98 (0.96)	0.098	0.89 (1.26)	<0.001	1829 (90.5)	787 (88.5)	0.128
No	1.08 (1.03)		0.62 (1.11)		193 (9.5)	102 (11.5)	
Aware of food packaging labels							
Yes	0.98 (0.95)	0.015	0.87 (1.25)	0.686	1860 (92.0)	815 (91.7)	0.833
No	1.16 (1.11)		0.83 (1.30)		162 (8.0)	74 (8.3)	
Aware of self-health							
Yes	1.00 (0.96)	0.091	0.88 (1.26)	<0.001	1953 (96.6)	858 (96.5)	1.000
No	0.83 (0.95)		0.45 (1.00)		69 (3.4)	31 (3.5)	
Regular BP							
Yes	1.12 (1.01)	<0.001	1.08 (1.35)	<0.001	1337 (66.1)	520 (58.5)	<0.001
No	0.76 (0.83)		0.49 (0.94)		685 (33.9)	369 (41.5)	
Smoking							
Yes	0.97 (0.99)	0.496	0.88 (1.33)	0.780	552 (27.3)	262 (29.5)	0.247
No	1.00 (0.95)		0.86 (1.22)		1470 (72.7)	627 (70.5)	
Drinking							
Yes	0.95 (0.94)	0.088	0.92 (1.25)	0.068	726 (35.9)	381 (42.9)	<0.001
No	1.01 (0.97)		0.83 (1.25)		1296 (64.1)	508 (57.1)	
Betel-Nut chewing							
Yes	0.91 (0.90)	0.098	0.80 (1.31)	0.320	235 (11.6)	105 (11.8)	0.933
No	1.00 (0.97)		0.87 (1.25)		1787 (88.4)	784 (88.2)	

The regression model for the frequency of outpatient visits, health examinations, and whether to seek medical care from the same physician (Table 3) shows significant correlation with gender, age and education-level variables. As shown in the results, four variables which include exercise habits, dietary habits (a low salt, low sugar, and low fat diet), regular blood pressure measurement and drinking habits correlate significantly with healthcare utilization (*P* <0.05). When comparing the results with people who exercise regularly, those without regular exercise regimens are less likely to visit a physician, undergo health exams, and seek medical care for illness from the same physician (odds ratio,OR = 0.70, 95%CI = 0.58–0.83). The individuals who do not follow healthy eating habits are more likely to visit physicians when compared to those with healthy eating habits. The individuals who do not check their blood pressure regularly are less likely to visit physicians and undergo health exams as compared to those who have regular blood pressure assessment. Those who drink regularly are less likely to seek medical care for illness from the same physician than those who do not drink (odds ratio,OR = 0.71, 95%CI = 0.58–0.85). The results of collinearity diagnosis reveal that the severity of association among all independent variables do not reach a significant level to affect the statistical computation.

**Table 3:** The Regression Model of Healthcare Utilization

Variable	Number of outpatient visits ^a^	Number of health exams ^b^	Seen by the same physicians
	B (95%CI)	B (95%CI)	OR (95%CI)
Exercise Habits			
No	* −0.39 (−0.67, −0.11)	** −0.24 (−0.33, −0.14)	**0.70 (0.58,0.83)
Breakfast			
No	0.06 (−0.74,0.87)	0.01 (−0.26,0.27)	1.06 (0.64,1.74)
Regular meals			
No	−0.06 (−0.76,0.64)	−0.03 (−0.26,0.20)	0.72 (0.47,1.09)
5 servings of fruits& vegetables			
No	0.46 (−0.01,0.92)	−0.06 (−0.21,0.10)	1.08 (0.80,1.44)
Dieting Habits			
No	*0.53 (0.02,1.53)	−0.03 (−0.19,0.14)	0.92 (0.67,1.25)
Aware of food packaging labels			
No	−0.34 (−0.89,0.21)	−0.05 (−0.23,0.14)	0.87 (0.62,1.23)
Aware of self-health			
No	−0.12 (−0.91,0.68)	−0.08 (−0.34,0.19)	1.41 (0.85,2.32)
Regular BP checks			
No	** −0.69 (−0.98, −0.38)	** −0.31 (−0.41, −0.21)	0.95 (0.79,1.14)
Smoking			
Yes	0.24 (−0.14,0.61)	−0.09 (−0.21,0.03)	1.05 (0.82,1.32)
Drinking			
Yes	−0.01 (−0.32,0.29)	0.09 (−0.01,0.19)	**0.71 (0.58,0.85)
Betel-Nut chewing			
Yes	−0.30 (−0.77,0.18)	0.002 (−0.15,0.16)	1.27 (0.94,1.70)

Data of gender, marital status, age, education levels, and diseases were adjusted

*p < 0.05; **p < 0.001

^a^ *R* ^2^ = 0.196

^b^ *R* ^2^ = 0.136

## Discussion

This survey consists of various aspects of health behaviors in daily life and detailed information of the utilization of preventive health care resources, which may complete the perspective of the main issues that this study would like to address. Regarding the correlation between health behaviors and the utilization of health care resources, the univariate analysis indicates that 6 of the 11 health behaviors are associated with the number of health exams; most of them are positively correlated, indicating that those individuals who incorporate healthy behaviors in their daily lives undergo health examinations more frequently. Although only four health behaviors are related to the other preventive healthcare behaviors (i.e., seeking medical care from the same physician), people in this population who incorporate healthy behaviors in their daily lives show a higher probability of seeing the same doctor. Additionally, four health behaviors correlate with the frequency of outpatient visits as well. However, having healthy behaviors might not definitely lead to a reduced frequency of outpatient visits. People who regularly exercise and measure their blood pressure appear to go to outpatient visits more frequently. After the adjustment of confounding variables, the results of the multivariate analysis show that there is a significant correlation between four health behaviors (i.e.,—having regular exercise habits, measuring blood pressure regularly, maintaining a low salt/low sugar/low fat diet, and having drinking habits) and the utilization of health care resources.

Regarding the utilization of preventive health care resources, individuals with a habit of exercise and performing blood pressure checks regularly have a higher tendency to undergo health exams, which corresponds with previous findings [22]; those who do not have drinking habits and those who have regular exercise regimens are more likely to seek medical care for illness from the same physician. Such results correspond to the findings of Qi, Phillips and Hopman (2006) [22]. The results demonstrate that the increased frequency of undergoing health exams and seeking the same physician for medical care may come with practicing healthy behaviors. Individuals require a remarkable level of motivation for self-health maintenance to continue regular daily exercise, blood pressure checks, and refrain from drinking. People who have a high motivation for keeping themselves healthy may discipline themselves to exercise and measure their blood pressure regularly, and to not consume alcohol. Also, these people understand the importance of undergoing health exams and seeking the same physician for medical care, and are thus more likely to perform these health behaviors and habits.

Additionally, there is a significant correlation between three health behaviors (i.e., having regular exercise habits, measuring blood pressure regularly,and maintaining a low salt/low sugar/low fat diet) and the frequency of outpatient visits. People who emphasize low salt, low sugar and low fat diets have fewer outpatient visits, which corresponds to the findings by Grisolia’s (2013) [23]. However, having healthy behaviors might not definitely lead to a reduced frequency of outpatient visits. The individuals with regular exercise habits and blood pressure checks appear to have more outpatient visits. The people who exercise regularly might receive better overall long term health benefits [19, 22, 24]. Several researchers also claim that the warm-up and stretching movements is highly important prior to the actual exercise in order to avoid sports-related injuries [25–28]. People with sports-related injuries will look for rehabilitative treatments, and may lead to an increased number of outpatient visits. This study did not thoroughly investigate the types and intensity of exercise, hence it is uncertain that exercise definitely causes injuries; the answers are pending future research and discussion. In addition, people who regularly measure blood pressure might not be used to perform this measurement by themselves due to their unfamiliarity with the operation of the machine, thus, they may opt for outpatient consultation as an alternative and lead to an increased utilization of this health care service [22].

Besides health behaviors, gender, age, educational level, and health status have also been found to have a correlation with the utilization of health care resources. This study shows that women visit doctors more often and are more willing to go to the same physician for an illness, which corresponds to the findings of Middleton, Hing and Xu (2013) [29]. Fewer women receive health examinations than men, possibly because of the low percentage of women in the job market. Employers are legally required to provide health examination services to employees within the workplace, and because the employment rate for women is low, the chance of them receiving health examinations is reduced proportionally [30, 31]. It is commonly hypothesized that the older the age, the poorer the health status, hence the frequency of outpatient visits and health exams is increased [32, 33]. The health policy in Taiwan provides regular health exam services for the middle-aged to senior population, increasing the opportunity for senior citizens to receive health examinations. There is a greater possibility for elderly people to seek medical care from the same physician when becoming ill [34]. The results of the multivariate analysis show that education levels have different effects on the frequency of outpatient visits and health exams. The welfare system of health insurance in Taiwan provide free health exams for a certain population and free oupatient visits for people with low social economical status. The results of this study show that the group with lower education levels has more outpatient visits and fewer health exams and is less likely to visit the same physician for illness. A possible reason is the lack of awareness of and demand for preventive healthcare, resulting in less desirable outcomes when health examinations are not obtained and not seeking medical care from the same physician. Most people with a lower educational level might also be categorized into people with lower SES (social economic status). People with lower SES have more privileges in terms of receiving health care services, however, people with a lower educational level, have limited knowledge of health care, that might have a negative effect on their health, and may result in an increased frequency of outpatient visits [22, 35–39]. The individuals suffering from disease have poorer health conditions, their number of doctor visits is higher compared with that of the individuals without diseases and they prefer to be seen by the same physician, which corresponds with previous findings [9]. Only the results from the univariate analysis showed correlations between marital status and the utilization of medical resources, although such correlations disappeared when confounding variables were controlled. Further research and validation are needed to understand the relationships between the variables. The limitation of this study is that the survey was only conducted in a regional city and the results of this study might not be able to generalize the trends as a global phenomenon.

## Conclusion

This study was conducted to understand whether people with healthy behaviors have reduced utilization of health care resources. Results show that people with healthy behaviors have increased utilization of preventive healthcare services, that they undergo health exams more frequently and have a higher probability of seeking the same physician for medical care as well. However, thedecrease in the number of outpatient visits is not that significant in people with healthy behaviors. Specifically, people with regular exercise habits and with regular blood pressure measurement do outpatient visits more frequently. In Taiwan, free health exams are only provided once a year or once every 3 years. People should continue incorporating healthy behaviors in their daily routines in order to maintain their health. One can say that the preventive health care resources that are provided by the government have been obviously insufficient. It is strongly suggested that health consultation centers shall be set up in strategic places nationwide to ensure the proper delivery of health-related services (i.e., public education, basic BMI/blood pressure/blood sugar/heart beat check-up, and consultation). Thus, people will be well-educated on the inculcation of healthy behaviors, the development and maximization of health care resources could be promoted, utilization of medical resources could be reduced and controlled, and lastly, the goal of public health may be achieved.

## Abbreviations

ANOVA: Analysis of variance
OR: Odds ratio
SPSS: Statistical Product and Service Solutions
